# Comparing methods for immobilizing HIV-1 SOSIPs in ELISAs that evaluate antibody binding

**DOI:** 10.1038/s41598-022-15506-x

**Published:** 2022-07-01

**Authors:** Kim-Marie A. Dam, Patricia S. Mutia, Pamela J. Bjorkman

**Affiliations:** 1 Division of Biology and Biological Engineering, California Institute of Technology, Pasadena, CA USA; 2 Division of Engineering and Applied Science, California Institute of Technology, Pasadena, CA USA

**Keywords:** Viral proteins, Assay systems

## Abstract

Enzyme-linked immunosorbent assays (ELISAs) are used to evaluate binding of broadly neutralizing antibodies (bNAbs) and polyclonal sera to native-like HIV-1 Env SOSIPs. Methods for immobilizing SOSIPs on plates differ, which can lead to variable or, in some cases, misleading results. Three methods used to immobilize SOSIPs were compared to determine how antigen immobilization methods affect Env conformation and ELISA results. HIV-1 SOSIPs were directly coated on polystyrene plates, captured by a monoclonal antibody against a C-terminal affinity tag, or randomly biotinylated and coated on a streptavidin plate. Binding of bNAbs with known epitopes were compared for each immobilization method. Binding of bNAbs targeting the V1V2, V3, CD4 binding site, and gp120/gp41 interface was comparable for all antigen immobilization methods. However, directly coated HIV-1 SOSIP ELISAs showed detectable binding of 17b, a CD4-induced antibody that binds a V3 epitope that is concealed on closed prefusion Env trimers in the absence of added CD4, whereas antibody-immobilized and randomly biotinylated Env-coated ELISAs did not show detectable binding of 17b in the absence of CD4. We conclude direct coating of HIV-1 SOSIPs on ELISA plates can result in exposure of CD4-induced antibody epitopes, suggesting disruption of Env structure and exposure of epitopes that are hidden in the closed, prefusion trimer.

## Introduction

**Figure 1:**
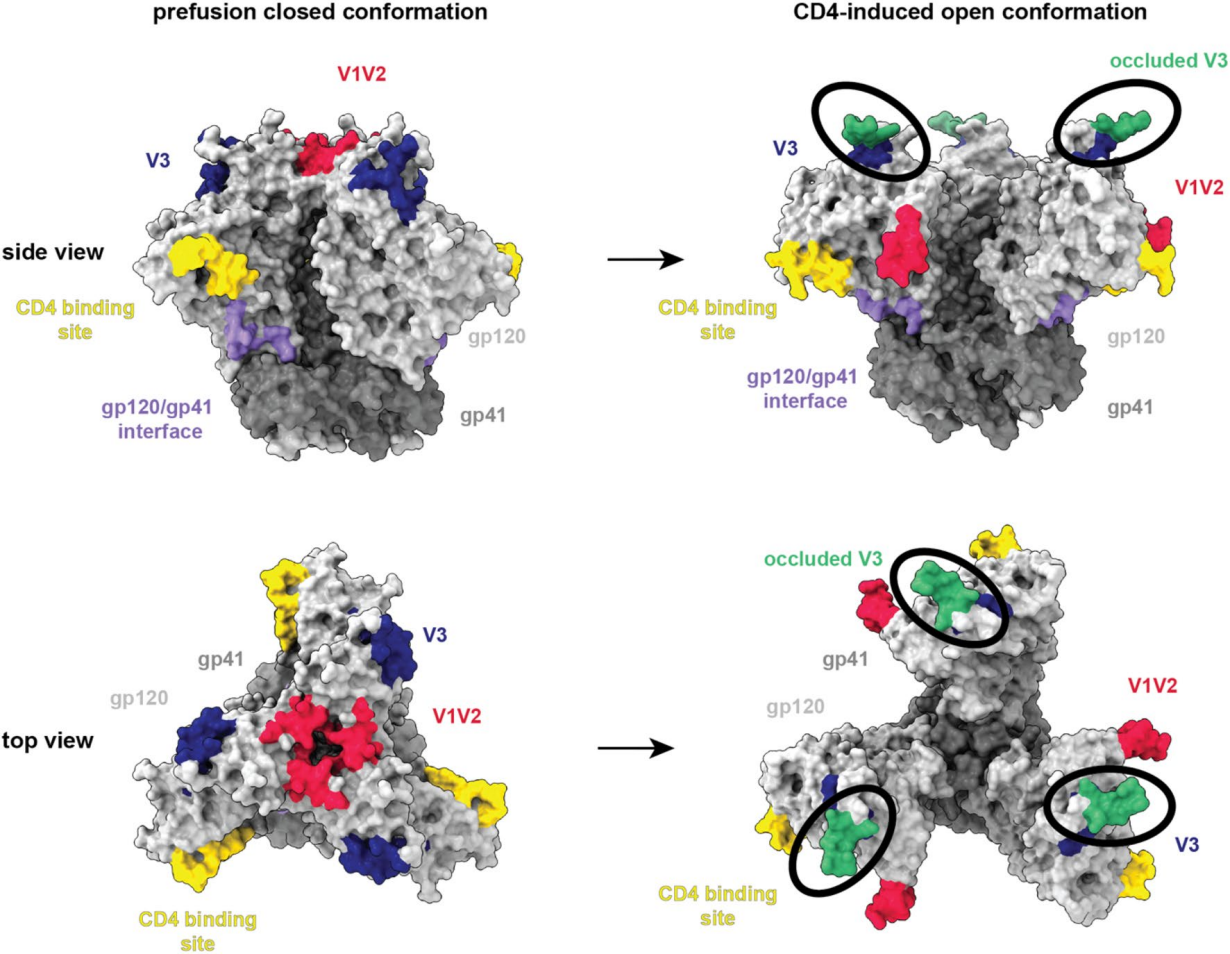
Summary of HIV-1 antibody binding sites on closed and CD4-induced open Env conformations. Surface depictions of HIV-1 Envs in closed and open conformations are shown from the side (top row) and from the top (bottom row). Left: HIV-1 bNAbs target epitopes on the closed, prefusion Env conformation (PDB 5T3Z) including V1V2 (crimson) (e.g., PG9), V3 (blue) (e.g., 10–1074), the CD4 binding site (yellow) (e.g., 3BNC117), and the gp120-gp41 interface (purple) (e.g., 8ANC195). Right: Upon interaction with CD4, Env undergoes conformational changes, including V1V2 displacement and exposure of the V3 base that is occluded in the closed conformation. The CD4-induced, open Env (PDB 5VN3) conformation is depicted with the occluded V3 regions (sea green) indicated in black ovals. 17b is an example of antibody that binds the occluded V3 epitope when Env is in the CD4-induced, open conformation. This figure was generated using UCSF ChimeraXv1.2.5^32,33^.

In 2020, 38 million individuals were living with human immunodeficiency virus-1 (HIV-1), the causative agent of acquired immunodeficiency syndrome (AIDS) (unaids.org). Despite 40 years of effort, a successful vaccine to prevent HIV-1 infection has not been developed. Current work towards vaccine design has focused on understanding how rare cases of natural infection induce broadly neutralizing antibodies (bNAbs) and how such antibody responses could be elicited by vaccination^1,2^. Efforts towards designing bNAb-inducing immunogens are focused on the closed, prefusion state of the HIV-1 Envelope (Env) trimer, the sole viral protein on the virion surface, which interacts with host cell receptors leading to viral entry into host cells^3–6^. The HIV-1 Env is a homotrimer composed of heavily-glycosylated gp120-gp41 heterodimers. To gain entry into cells, the Env gp120 subunit binds host CD4 receptors causing conformational changes that lead to virus and host cell membrane fusion and viral entry. These changes have been structurally characterized and include displacement of the gp120 V1V2 loops to expose occluded V3 loops and the CCR5/CXCR4 co-receptor binding site resulting in CD4-bound “open” trimers (Fig. 1)^7–9^.

Broadly neutralizing antibodies (bNAbs) have been isolated from a subset of HIV-1–infected donors and target conserved epitopes of the HIV-1 Env with exceptional breadth and potency^10^. bNAbs have been characterized to contain uncommon features that have been found necessary to accommodate the dense glycan shield concealing conserved Env epitopes and are elicited only in rare cases of natural infection^2,10^. Most bNAbs target epitopes on the closed prefusion HIV-1 Env conformation, although more strain-specific antibodies have been found to bind epitopes that are hidden on closed, prefusion Env trimers that are usually only exposed after CD4 binding (Fig. 1)^7,11,12^.

A soluble form of the trimeric Env ectodomain, called Env SOSIP^13^, was designed with stabilizing mutations to favor the closed, prefusion Env conformation targeted by bNAbs and to allow Envs to be studied biochemically and structurally and used as immunogens. Structural biology and DEER spectroscopy have characterized the conformational profiles of soluble SOSIP Env proteins, finding that unliganded SOSIP trimers adapt a prefusion, closed conformation and that interactions of SOSIP trimers with soluble CD4 (sCD4) leads to the above described conformational changes (Fig. 1)^7,8,10,14,15^. Both the closed prefusion and CD4-induced open SOSIP conformations are consistent with cryo-electron tomography and sub-tomogram averaged structures of HIV-1 Envs found on virions^16–20^. Although SOSIP Envs can adopt both open and closed trimer conformations, HIV-1 immunogen design efforts seek to raise bNAbs against closed prefusion Envs, thus methods to evaluate antibody binding to SOSIPs should maintain the closed Env conformation in the absence of added sCD4.

Enzyme-linked immunosorbent assays (ELISAs) are routinely used to characterize bNAb binding and evaluate polyclonal serum responses for vaccine development. In an ELISA, the soluble form of the HIV-1 Env SOSIP is generally immobilized on the surface of the assay plate, and then bNAbs or sera are added and detected through a secondary antibody conjugated to an enzyme capable of a colorimetric readout in the presence of its substrate. Since bNAbs target the closed, pre-fusion conformation of Env, it is essential that Env-coating methods in an ELISA do not alter or disrupt the native-like closed prefusion Env conformation or binding to normally hidden epitopes would be observed. ELISAs that evaluate antibody binding to antigens often involve direct addition of antigen to an ELISA plate, where it associates with the plastic wells in an unknown manner. Although association with plastic likely does not affect the conformation of most antigens, the ability of HIV-1 SOSIP Env trimers to undergo large conformational changes could cause SOSIPs to adopt different conformations when associating with plastic wells. In addition, the dense glycan shield on HIV-1 Envs, which varies between strains, could cause strain-specific differences in how SOSIPs adhere to plastic that could affect binding of some types of antibodies.

To better understand how Env immobilization affects bNAb binding, we compared direct coating of SOSIP Envs on ELISA plates with other attachment methods that avoid direct contact of HIV-1 SOSIP Envs with plastic. In the preferred method, a peptide tag is added to the SOSIP C-terminus, allowing site-specific biotinylation or capture by an antibody for oriented coupling to an ELISA plate^13,21^. Because terminally-tagged proteins can lose their tags due to proteolysis upon storage (unpublished observations) and/or are not always available for ELISAs involving multiple SOSIPs, we tried a method that would allow already-prepared untagged SOSIPs to be used in an ELISA without direct coating onto plastic: attaching biotins randomly to primary amines on SOSIP Env and coating the randomly biotinylated SOSIPs on a streptavidin assay plate.

## Results

### Directly coated BG505 SOSIP Env ELISAs show detectable 17b binding in the absence of sCD4

**Figure 2:**
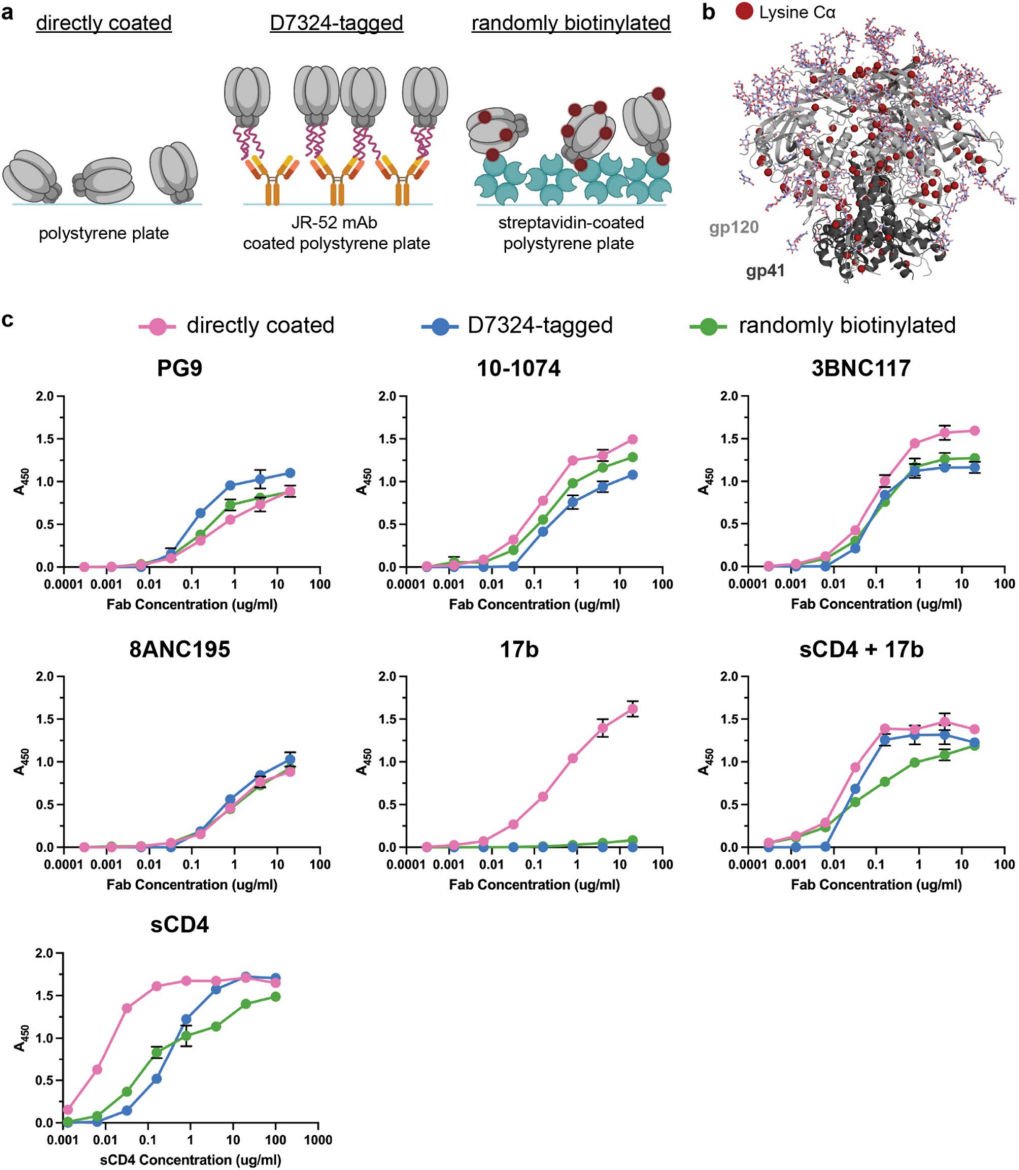
ELISA methods to immobilize BG505 SOSIP affect binding of the CD4-induced 17b antibody. (**a**) Representation of ELISA methods to immobilize the HIV-1 Env antigen include directly coating the Env on polystyrene plates, capturing Env that contains a D7324 affinity tag with the mAb, JR-52, and randomly biotinylating Env to immobilize on streptavidin-coated plates. Created with BioRender.com. (**b**) Structure of HIV-1 Env (PDB 5T3Z) with the locations of primary amine lysine carbon-alpha atoms (Cα) indicated as red spheres. The abundance of Env lysines allows for minimal disruption to the Env structure when biotinylating only 1–10 lysines per protomer. Created with The PyMolv2.2.3 Molecular Graphics System (Schrödinger, LLC). (**c**) ELISAs comparing binding of bNAbs and sCD4 for directly coated (pink), D7324-tagged (blue), and randomly biotinylated (green) BG505 Env immobilization methods. Values shown are means ± s.d. of two biological replicates (n = 2). Error bars are not visible for data points where the bars are smaller than the symbol representing the mean.

To evaluate how SOSIP Env antigen immobilization affects antibody binding in ELISAs, we compared three different methods for immobilizing HIV-1 Env antigens. SOSIP Envs were either directly coated onto polystyrene plastic wells, captured by a C-terminal D7324 affinity tag using the JR-52 mAb^13^, or randomly biotinylated and immobilized on streptavidin-coated wells (Fig. 2a). For randomly biotinylated SOSIP ELISAs, biotins were chemically attached to primary amines (lysine residues and N-termini). Since the biotinylation reaction does not go to completion, not all primary amines have attached biotins, resulting in SOSIPs with different sets of biotinylated lysines. For example, in BG505 SOSIP, we measured only 1–10 biotins per gp120-gp41 protomer, although each protomer contains 33 lysines (Fig. 2b). Since a random set of lysines are biotinylated on each protomer within a population of Env trimers, it is unlikely that particular lysines would be targeted among those that are accessible (Fig. 2b) or that particular antibody epitopes would be occluded within the overall population of Env trimers.

These SOSIP Env immobilization methods were used to compare binding of bNAbs to different epitopes on the clade A BG505 SOSIP trimer (Supplementary Table 1)^13^. We selected the following bNAbs against defined Env epitopes: PG9 against the V1V2 apex, 10–1074 against the V3 loop, 3BNC117 against the CD4 binding site, and 8ANC195 against the gp120-gp41 interface (Fig. 1, Supplementary Table 2). We chose the mAb 17b to assess conformational integrity of immobilized SOSIP Envs because it recognizes an occluded V3 epitope that is exposed only upon CD4 binding (Fig. 1, Supplementary Table 2)^7,8,22–25^. In the original characterization of the native-like BG505 SOSIP Env trimer, 17b showed binding only in the presence of sCD4, which demonstrated that it was properly folded and stabilized in the closed prefusion conformation^13^.

Binding for PG9, 10–1074, 3BNC117, and 8ANC195 was comparable among directly coated, D7324-tag immobilized, and randomly biotinylated BG505 SOSIP ELISAs (Fig. 2c). As expected, all ELISA methods showed binding for 17b in the presence of sCD4 (Fig. 2c). However, directly coated Env ELISAs showed substantial binding of 17b in the absence of sCD4, whereas D7324-immoblized and randomly biotinylated SOSIP ELISAs did not show binding for 17b alone (Fig. 2c). These results demonstrate that all ELISA coating methods allow detection of CD4-induced conformational changes, but that direct coating of the BG505 SOSIP on an ELISA plate inappropriately exposes the 17b epitope that is occluded on the stabilized closed, prefusion Env trimer structure.

### Multiple Env SOSIPs and SOSIP-based immunogens showed binding of 17b in the absence of sCD4 when directly coated on plastic

**Figure 3:**
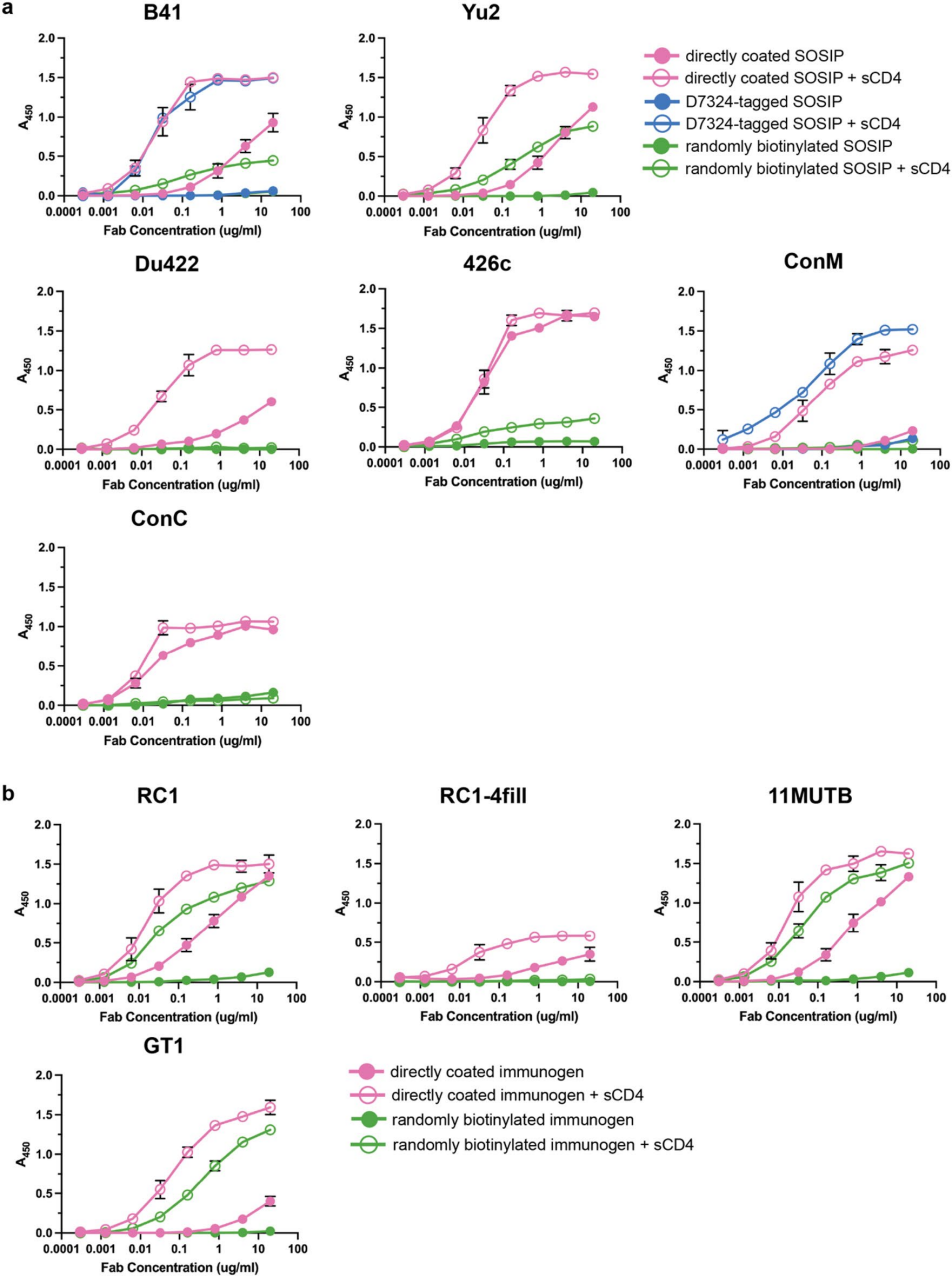
Directed coated ELISAs show 17b binding in the absence of sCD4 for multiple Env strains and Env-based immunogens. (**a**) ELISAs to compare 17b binding in the absence and presence of sCD4 to Clade B (B41, Yu2), Clade C (Du422, 426c) and consensus sequence (ConM and ConC) Envs for directly coated (pink), D7324-tagged (blue), and randomly biotinylated (green) Env immobilization methods. (**b**) ELISAs comparing 17b binding in the absence and presence of CD4 to V3-targeting (RC1, RC1-4fill, 11MUTB) and CD4bs (GT1) Env-based immunogens for directly coated (pink) and randomly biotinylated (green) Env immobilization methods. Values shown are means ± s.d. of two biological replicates (n = 2). Error bars are not visible for data points where the bars are smaller than the symbol representing the mean.

To investigate whether other SOSIP Env trimers exposed the 17b epitope in the absence of added sCD4, we evaluated 17b binding in the absence and presence of sCD4 against SOSIP Envs from diverse HIV-1 strains that were immobilized through direct coating, the D7324 tag, or random biotinylation (Fig. 3a). These included SOSIP Envs from clade B strains B41 and Yu2, clade C strains Du422 and 426c, and ConM and ConC Envs representing consensus sequence of group M and C Env isolates, respectively^26^. We observed 17b binding in all ELISAs in the presence of sCD4, as expected, although 17b binding in the presence of sCD4 was lower for randomly biotinylated SOSIPs compared to directly coated and D7324-tagged Env (Fig. 3a), demonstrating that all SOSIP trimers evaluated were able to undergo CD4-induced conformation changes. However, with the exception of ConM, directly coated SOSIP ELISAs showed 17b binding to SOSIPs in the absence of sCD4, whereas for D7324-tagged and randomly biotinylated Env, 17b binding only occurred in the presence of added sCD4. These results demonstrated exposure of the 17b epitope upon direct coating of most SOSIP Env trimers, thus suggesting that plastic-induced conformational changes in SOSIP Envs is a commonly-seen phenomenon that could affect interpretation of ELISA results.

An important application of Env antigen ELISAs includes evaluating binding of bNAbs, mAbs, and polyclonal serum from immunized animals against Env-based immunogens that include mutations intended to target or elicit particular bNAb precursors. These ELISAs have been used to characterize how antibodies elicited in vivo interact with immunogens. To determine if engineered Env-based immunogens are affected by Env-coating methods, we compared 17b binding in the absence and presence of sCD4 against known SOSIP Env-based immunogens. We chose the RC1, RC1-4fill, and 11MUTB immunogens that were developed to elicit V3 bNAb responses^27,28^ and have been tested in immunization regimens in animal studies (Supplementary Table 1)^29^. We also included BG505 SOSIP.v4.1-GT1 (GT1), an immunogen engineered to interact with the V1V2 apex and CD4-binding site bNAb precursors and currently being evaluated in human clinical trials (Supplementary Table 1)^30^. All immunogens included Env residue substitutions and the addition and/or deletion of certain Env N-linked glycan sites. Immunogens were either directly coated and immobilized on ELISA plates or randomly biotinylated and immobilized via streptavidin-coated plates. Consistent with previous observations for the unaltered “wildtype” SOSIP Env trimers, sCD4 bound to diverse Env strains (Fig. 3a) and Env-based immunogens (Fig. 3b) tested (Supplementary Fig. 1). However, once again, detectable 17b binding in the absence of sCD4 was observed for all directly coated Env-based immunogens and not for those that were randomly biotinylated.

## Discussion

The HIV-1 Env trimer is a highly evolved fusion machine that undergoes large conformational changes during host receptor engagement. Therefore, methods used to investigate binding properties of soluble Env SOSIPs commonly used as immunogens and to evaluate bNAb binding must ensure the native-like closed, prefusion Env trimer conformation is maintained in the absence of CD4 engagement. ELISAs are widely utilized in HIV-1 research to detect the of binding of bNAbs and polyclonal serum antibodies to Envs. Several methods have been used to immobilize SOSIP Envs on ELISA plates. In our comparison of three methods commonly used to immobilize HIV-1 SOSIP on ELISA plates and detect bNAb binding, we found that directly coating HIV-1 Env SOSIPs on plates leads to aberrant binding of the CD4-induced 17b antibody in the absence of sCD4. This suggests that the HIV-1 Env is not maintaining a prefusion, closed conformation representative of the closed, prefusion conformation of native Envs on virions^16–20^ and instead exposes a concealed V3 epitope found on sCD4-bound open Env trimer structures^7,8^. Exposure of the V3 epitope occurs when CD4 interacts with Env and causes conformational changes in the gp120 subunit leading to displacement of the V1V2 loops by ~ 40 Å from the trimer apex to the sides of the trimer^7,8^. 17b binding to directly coated SOSIP Env trimers in the absence of sCD4 addition suggests that interactions with plastic can disrupt the native-like closed, prefusion Env structure, leading to V3 exposure and subsequent binding of 17b. Therefore, directly coating SOSIPs on ELISA plates could lead to detection of binding of antibodies against occluded epitopes that would not normally recognize a closed, prefusion Env trimer, confounding interpretation of antibody binding and epitope mapping experiments. This would especially be problematic when using ELISAs for antibody epitope mapping studies in which mutations were introduced in defined Env epitopes, since the original and mutant SOSIPs could differ in their interactions with plastic. This could result in artifactually different antibody binding profiles.

The preferred methodology for SOSIP ELISAs is to use a C-terminal affinity tag capture immobilization so that the SOSIP Env does not interact directly with the plastic wells^13,21^. However, in some cases, an investigator may wish to compare antibody binding to a large number of already-prepared untagged SOSIP Envs. In this instance, random biotinylation and coating onto a streptavidin plate could be used instead of re-expression and purification of tagged SOSIP Env trimers. Since biotinylation methods can be optimized to biotinylate only a fraction of the primary amines (lysine side chains and the N-termini), it is unlikely that any particular antibody epitope would be occluded on all Env trimers within a randomly biotinylated population of SOSIPs, as demonstrated here for bNAb recognition of randomly biotinylated BG505 SOSIP. Importantly, in common with the C-terminal tag-immobilized SOSIPs, but not with directly coated SOSIPs, randomly biotinylated SOSIPs exhibited binding of the CD4-induced 17b mAb in the presence, but not the absence, of added sCD4. This suggested that proper folding of the closed, prefusion SOSIP Env trimer was maintained during an ELISA experiment involving randomly biotinylated SOSIPs. We note, however, that 17b binding in the presence of sCD4 was reduced in the randomly biotinylated SOSIPs compared with C-terminally tagged or directly coated SOSIPs, suggesting some interference with CD4-induced gp120 and gp41 conformational changes resulting from randomly biotinylation. Therefore, C-terminally tagged SOSIP Env trimers should be used for ELISA experiments involving quantitative assessment of sCD4-induced conformational changes but are not required for ELISAs evaluating antibodies binding to closed, prefusion Env trimers or non-quantitative ELISAs assessing binding to CD4-induced epitopes.

## Methods

### Protein expression and purification

The Expi293 transient transfection system (Thermo Fisher) was used to express Fabs and sCD4 as previously described^12^. The expression vectors for Fabs contained genes for a light chain (LC) and a C-terminally 6x-His tagged heavy chain (HC), and expression vectors for sCD4 encoded the D1D2 subunits of sCD4 followed by a C-terminal 6x-His tag. Fabs and sCD4 were purified from cell supernatants using a Ni^2+^-NTA (GE Healthcare) affinity chromatography column, followed by a Superdex 200 10 300 size exclusion chromatography (SEC) column (Cytiva).

HIV-1 SOSIP.664 Env constructs contained the following modifications: disulfide mutations 501C and 650C (SOS), I55P (IP), and the furin cleavage site mutated to six arginine residues (6R)^13^. D7324-tagged Env SOSIPs encoded a GSAPTKAKRRVVQREKR sequence after residue 664 in the gp41 ectodomain^13^. HIV-1 SOSIP.664 Env-based immunogens contained further mutations as described previously (Supplementary Table 1)^27–30^. Genes encoding all HIV-1 SOSIP.664-based constructs were expressed using the Expi293 transient transfection system (Thermo Fisher). All Envs were separated from cell supernatants using a 2G12 or PGT145 immunoaffinity chromatography column followed by SEC using a Superose 6 10/300 column (Cytiva) as described^13,31^.

### Enzyme-linked immunosorbent assay (ELISA)

HIV-1 SOSIP.664 trimer antigens were coated onto ELISA plates by three different methods. For directly coated antigen ELISAs, Corning Costar high-binding 96-well plates were coated with 5 µg/mL of a SOSIP diluted in 0.1 M NaHCO_3_ (pH 9.6) and incubated overnight at 4 °C. Unbound trimers were removed by washing, and plates were blocked with 3% BSA in TBS-T (20 mM Tris, 150 mM NaCl, 0.1% Tween20) for 1 h at room temperature, and then buffer was removed. For D7324-capture ELISAs involving immobilization of D7324-tagged SOSIPs to the JR52 mAb^13^, JR-52 (kind gift from James Robinson, Tulane University) was coated on Corning Costar high-binding 96-well plates in 0.1 M NaHCO_3_ (pH 9.6) and incubated overnight at 4 °C^13^. Excess JR-52 was removed, plates were blocked for one hour at room temperature in 3% BSA in TBS-T, and blocking buffer was removed. D7324-tagged Envs were added at 5 µg/mL in 3% BSA in TBS-T and incubated at room temperature for one hour before buffer was removed. For randomly biotinylated antigen ELISAs, Env trimers were randomly biotinylated using the EZ-Link NHS-PEG4-Biotin kit (Thermo Fisher Scientific) according to the manufacturer’s guidelines. The Pierce Biotin Quantitation kit (Thermo Fisher Scientific) was used to quantify the number of biotin molecules per Env protomer, resulting in an average of 1–10 biotins attached to each Env protomer. Randomly biotinylated Envs were added to streptavidin-coated 96-well plates (Thermo Fisher Scientific) at a concentration of 5 µg/mL diluted in 3% BSA in TBS-T. Plates were incubated for two hours, and then access antigen was removed.

For some experiments, sCD4 was added at 100 µg/mL and incubated at room temperature for two hours. bNAb Fabs or sCD4 were serially diluted in 3% BSA in TBS-T at a top concentration of 20 µg/mL and 100 µg/mL, respectively, and then incubated for two hours at room temperature. Fabs and sCD4 were removed and plates were washed twice with TBS-T. Horseradish peroxidase (HRP) labeled secondary against the human IgG H + L (Southern BioTech) added at 1:10,000 dilution in TBS-T to detect Fab binding or HRP labeled secondary against the 6x-His tag (Genscript) added a 1:5000 dilution in TBS-T to detect His-tagged sCD4 were added and incubated at room temperature for 30 min. Plates were then washed three times with TBS-T. Ultra TMB-ELISA Substrate Solution (ThermoFisher Scientific) was added for colorimetric detection and quenched with 1.0 N HCl. Absorption was measure at 450 nm. Two independent biological replicates (n = 2) were performed for all assays.

## Supplementary Information

Supplementary Information.

## Data Availability

The datasets generated during and analyzed in this study are available from the corresponding author on reasonable request.
