# Supplementary material for: Comparing methods for immobilizing HIV-1 SOSIPs in ELISAs that evaluate antibody binding
Source: Sci Rep. 2022 Jul 1;12:11172. doi: 10.1038/s41598-022-15506-x (PMC9247892; doi:10.1038/s41598-022-15506-x)
Supplement: Supplementary file 1 — Supplementary Information. [file 41598_2022_15506_MOESM1_ESM.docx]

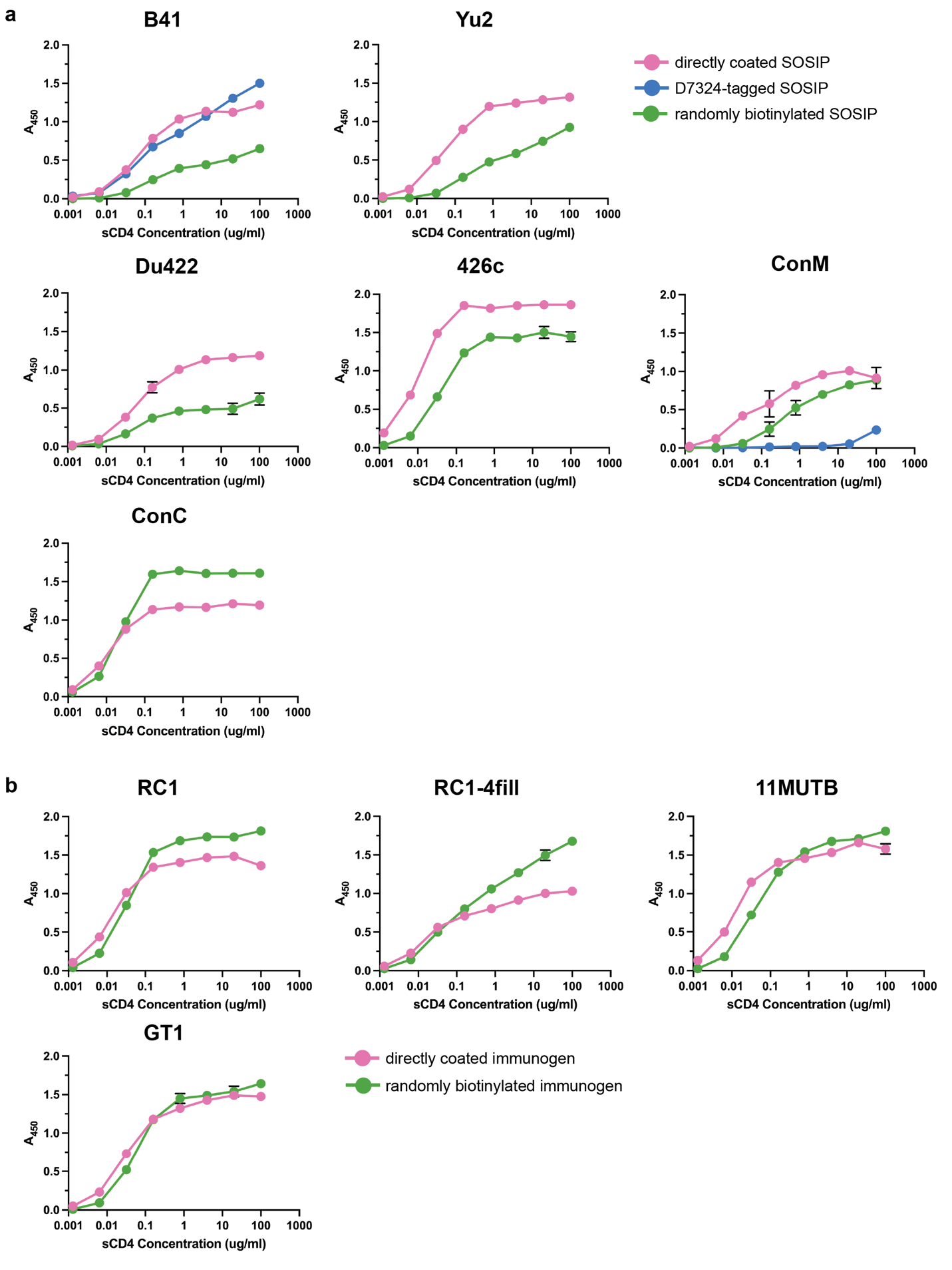


**Supplementary Figure 1: All Env coating ELISA methods lead to sCD4 binding**

(**a**) ELISAs to compare sCD4 binding to Clade B (B41, Yu2), Clade C (Du422, 426c) and consensus sequence (ConM and ConC) Envs for directly coated (pink), D7324-tagged (blue), and randomly biotinylated (green) Env immobilization methods. (**b**) ELISAs comparing binding of CD4 to V3-targeting (RC1, RC1-4fill, 11MUTB) and CD4bs (GT1) Env-based immunogens for directly coated (pink) and randomly biotinylated (green) Env immobilization methods. Values shown are means ± s.d. of two biological replicates (n=2). Error bars are not visible for data points where the bars are smaller than the symbol representing the mean.

**Supplementary Table 1: Summary of HIV-1 Env SOSIPs used in ELISA experiments**

| **Strain** | **Clade** | **Tier** | **Mutations** | **Reference** |
| --- | --- | --- | --- | --- |
| BG505 | A | 2 | SOSIP (501C-605C, I559P, R6); added PNGS: 332 | Sanders, et al., *PLOS Pathog.,* (2013) |
| B41 | B | 2 | SOSIP (501C-605C, I559P, R6) | Pugach, et al., *J. Virol*, (2015) |
| Yu2 | B | 2 | SOSIP (501C-605C, I559P, R6) | Dosenovic, et al., *Cell*, (2015) |
| Du422 | C | 2 | SOSIP (501C-605C, I559P, R6) | Julien, et al., *PNAS*, (2015) |
| 426c | C | 2 | SOSIP (501C-605C, I559P, R6) | Borst, et al., Elife, (2018) |
| ConM | N/A | 1A | SOSIP (501C-605C, I559P, R6) | Sliepen, et al., *Nat. Commun.,* (2019) |
| ConC | N/A | unknown | SOSIP (501C-605C, I559P, R6) | Rutten, et al., *Cell Rep.,* (2018) |
| RC1 | A | N/A | SOSIP (501C-605C, I559P, R6); added PNGS: 332; point mutations: V134Y, N136P, I138L, D140N, N137F, T320F, Q328M, T415V; PNGS deletions: N133, N137, N156 | Escolano*, Gristick*, et al., *Nature*, (2019) |
| RC1-4fill | A | N/A | SOSIP (501C-605C, I559P, R6); added PNGS: 230, 241, 289, 332, 344; PNGS deletions: N133, N137, N157; point mutations: V134Y, N136P, I138L, D140N, N137F, T320F, Q328M, T415V | Escolano*, Gristick*, et al., *Nature*, (2019) |
| 11MUTB | A | N/A | SOSIP (501C-605C, I559P, R6); added PNGS: 332; PNGS deletions: N133, N137; point mutations: V134Y, N136P, I138L, D140N, N137F, T320F, Q328M, T415V | Steichen, et al., *Immunity*, (2016) |
| GT1 | A | N/A | SOSIP (501C-605C, I559P, R6); added PNGS: 332; PNGS deletions: N197, N276, N462; point mutations: K169R, Y173H, S174A, R179K, V181I, Q183P, G188N, N189T, E190S, delR_185f_SNNSNK_189_, T278R, G471S | Medina-Ramírez, et al., *JEM*, (2017) |

*Indicates authors contributed equally

**Supplementary Table 2: Summary of antibodies used in ELISA experiments**

| **mAb** | **Epitope** | **PDB accession code** | **Reference** |
| --- | --- | --- | --- |
| PG9 | V1V2 | 5VJ6 | Wang, et al., *Elife,* (2017) |
| 10-1074 | V3 | 5T3Z | Gristick, et al., *NSMB,* (2016) |
| 3BNC117 | CD4 binding site | 5V8M | Lee, et al., *Immunity*, (2017) |
| 8ANC195 | gp120/gp41 interface | 5VJ6 | Wang, et al., *Elife,* (2017) |
| 17b | V3 base | 5VN3 | Ozorowski, et al., *Nature*, (2017) |
